# Optimization of the Extraction Conditions of Polyphenols from Red Clover *(Trifolium pratense* L.) Flowers and Evaluation of the Antiradical Activity of the Resulting Extracts

**DOI:** 10.3390/antiox13040414

**Published:** 2024-03-28

**Authors:** Beata Drużyńska, Jakub Łukasiewicz, Ewa Majewska, Rafał Wołosiak

**Affiliations:** Institute of Food Sciences, Department of Food Technology and Assessment, Division of Food Quality Assessment, Warsaw University of Life Sciences (WULS-SGGW), 159C Nowoursynowska Street, 02-776 Warsaw, Poland; p000622@wp.pl (J.Ł.); ewa_majewska1@sggw.edu.pl (E.M.); rafal_wolosiak@sggw.edu.pl (R.W.)

**Keywords:** red clover, polyphenols, antioxidant activity, HPLC, optimization, extraction conditions

## Abstract

The purpose of this study was to analyze the effect of the type of extraction solution (water, different concentrations of ethanol), temperature and time on the polyphenol content and antioxidant properties of red clover extracts and the effect of the addition of selected extracts on the antioxidant properties of enriched blackcurrant beverages. In both the extractions carried out under different conditions and in the enriched beverages, the content of selected polyphenols was determined by HPLC. This study confirmed the significant effect of the alcohol content of the extract, extraction time and temperature on the antioxidant properties of clover extracts. Ethanolic extracts had better antioxidant properties than aqueous extracts. The addition of ethanol extracts had a significant effect on the antioxidant properties of the fortified beverages. Increasing the temperature, time or ethanol content in the extracts mostly resulted in an increase in the total polyphenol content in the obtained extracts. Based on the analysis of the response surface, it was found that for the DPPH radical, the best activity was obtained by extraction for 20 min with a solution of approximately 65% at low temperatures. In the case of the ABTS radical, the best antiradical activity was obtained after extraction for 60 min at 80 °C with a solution of approximately 50% ethanol. It was also found that the use of a solution of approximately 60% ethanol after extraction for 60 min at 80 °C would provide an extract with high antiradical activity against both radicals.

## 1. Introduction

Red clover (*Trifolium pratense*) belongs to the bean family (*Leguminosae*). It is a leguminous and perennial plant that grows in temperate and humid climates. It reaches a height of 10–15 cm, has oval three-leaved leaves and usually lasts from 3 to 5 years. The plant belongs to the genus *Trifolium* species (about 250 species) [1,2,3].

The cultivation of clover dates back to the 3rd century AD, and today it is grown to increase soil fertility, which was used as early as the 16th century. In addition, red clover is used in animal feed. This is due to its high protein content and low soluble fiber fraction. It owes its high protein content to its microflora, which includes bacteria capable of fixing atmospheric nitrogen and their symbiosis with the soil microflora. Red clover occurs naturally in Europe, West Asia and northwest Africa, but is grown in many parts of the world due to its high adaptability to different soils [2,4,5].

The primary compounds responsible for the antioxidant properties of clover are polyphenols. These chemical compounds, abundantly present in plants, exhibit notable antiradical characteristics. In the human diet, polyphenols act as antioxidants, helping to fight free radicals and reduce the risk of many diseases, including those of civilization, such as heart disease, cancer and neurodegenerative diseases [6,7].

There are many polyphenols in clover. These primarily include isoflavones such as biochanin A, formononetin, daidzein and genistein, and flavonoids such as quercetin and kaempferol. Phenolic acids such as caffeic acid, gallic acid and chlorogenic acid can also be found in red clover. Isoflavones, whose structure is based on phenylbenzopyran, are found most in clover. They are stored in the plant in conjugated form as O-glycosides. Their composition, content and estrogenic activity in clover depend on the time and conditions of growth. The highest content of isoflavones can be obtained when clover is grown using white light at 25 °C for 10 days after germination [8,9,10].

Studies indicate that isoflavones attach to estrogen receptors, reducing the proliferative properties of estrogen. These receptors come in two forms: α and β. Isoflavones attach to both forms, but their affinity for the ß form is greater. Attachment of compounds to β receptors decreases the activity of α receptors. α receptors increase proliferation properties after estrogen attachment, resulting in an increased risk of osteoporosis and cardiovascular disease. Decreasing the activity of α receptors results in a decreased risk of osteoporosis and related diseases and cardiovascular diseases without the risk of hormone-initiated cancer [11,12].

Due to its high content of estrogenic isoflavones, red clover has the ability to reduce symptoms of estrogen deficiency. Reactions of isoflavones with estrogen receptors help reduce estrogen deficiencies that occur in women’s bodies during and after menopause. Supplementation with isoflavones found in red clover helps reduce the risk of osteoporosis and related diseases, as well as cardiovascular disease [2,9].

Red clover has been used in folk and traditional medicine. Its extracts are still used in the treatment of menopausal symptoms, as an antiradical and anti-aging agent, in the treatment of hyperlipidemia and metabolic syndrome, to strengthen the cardiovascular system, to prevent climacteric osteoporosis and benign prostatic hyperplasia and experimentally in the treatment of prostate cancer [2,13]. Currently, red clover is mainly used as feed for ruminants due to its high fiber content. It is characterized by a high protein content, which is 15–30% of dry matter, depending on the harvest season. Red clover, along with white clover, is also used to increase soil fertility by simultaneously introducing microorganisms capable of fixing atmospheric nitrogen, which naturally occur in various parts of these plants [14,15,16].

Red clover is used in supplementation to reduce symptoms of estrogen deficiency. Supplements containing red clover extracts are also used to treat respiratory, nervous and reproductive diseases [9,17]. Clover contains polyphenol oxidase, which, along with a protein found in the plant, can be used to secure polyunsaturated fatty acids in emulsions. The presence of polyphenol oxidase results in the formation of protein–phenol complexes. Fatty acids are encapsulated in these complexes, which increases their oxidative stability. The possibility of using red clover extract for this purpose was studied by Gadeyne et al. [18]. Emulsions obtained from red clover protein extract had higher oxidative stability compared to casein emulsions. Thus, the plant has great potential both as an ingredient in the daily diet, but also, potentially, as an agent capable of counteracting the oxidation of food ingredients in industrial technology.

The purpose of this study was to evaluate the effect of different extraction conditions (water, ethanol at different concentrations, time, temperature) of polyphenolic compounds from red clover (*T. pratense* L.) on the antiradical properties and content of these compounds in the extracts obtained, as well as blackcurrant beverages enriched in selected extracts.

## 2. Materials and Methods

### 2.1. Materials

For the test, powdered dried red clover (*Trifolium pratense* L.) was purchased from an online store (MyVita^®^, Poland). During the test, it was stored in the original packaging in a dry and dark place. Six blackcurrant drinks from the same company (Tymbark, Krakow, Poland) and batch were also purchased from a grocery store in 300 cm^3^ bottles. The beverage bottles were stored in a dark place until analysis. The beverage bottles were opened immediately before enrichment with the appropriate extracts.

### 2.2. Methods

#### 2.2.1. Extraction

The extracts were prepared by weighing 10.0 g each of red clover powder on an analytical balance into 300 cm^3^ conical flasks and adding each 100 cm^3^ of extraction solution, which were water, 40% ethanol solution (*v*/*v*), 60% ethanol solution (*v*/*v*) and 80% ethanol solution (*v*/*v*) (Pol-Aura, Zabrze, Poland). The extracts prepared in this way were subjected to shaking on a shaker for 20, 40 and 60 min and using temperatures of 20 °C, 40 °C and 80 °C. Then, the extracts were filtered into conical flasks, covered with stoppers and protected with parafilm. The extracts were stored for further analysis in the freezer for no longer than 4 weeks.

#### 2.2.2. Beverage Preparation

The beverages were prepared by adding 2% and 5% (*v*/*v*) aqueous and alcoholic extract, which showed the best antiradical properties in earlier determinations (extraction with 80% EtOH, temp. 80 °C, time 40 min). Beverages were stored in the freezer for further analysis for no longer than 4 weeks. The samples were labeled as follows: C—blackcurrant beverage control sample, EW2—beverage enriched with 2% aqueous extract, EW5—beverage enriched with 5% aqueous extract, EEtOH2—beverage enriched with 2% ethanol extract, EEtOH5—beverage enriched with 5% ethanol extract.

#### 2.2.3. Determination of the Content of Total Polyphenols

The quantification of total polyphenols was conducted employing the Folin–Ciocalteu method [19]. To elaborate, 300 μL aliquots of either ethanol (varying concentrations: 20%, 40%, 60%) or water extracts were dispensed into test tubes. Subsequently, 4.15 mL of deionized water, 500 μL of a 20% Na_2_CO_3_ solution (Pol-Auta, Zabrze, Poland) and 50 μL of Folin–Ciocalteu (Pol-Aura, Zabrze, Poland) reagent were introduced into each tube. The absorbance was then gauged after a 20 min incubation period at a wavelength of 760 nm (utilizing a Shimadzu UV-1201V spectrophotometer (Shimadzu Corporation, Kyoto, Japan). The total polyphenol content was determined utilizing the established standard curve and was expressed as gallic acid equivalent (GAE) per 100 g of dry matter. A series of gallic acid standards (Sigma Aldrige, Poznań, Poland) were prepared through dilution with varying volumes (0 mL, 1 mL, 2 mL, 3 mL, 5 mL, 10 mL and 20 mL) of gallic acid stock solution into six 100 mL volumetric flasks, which were then made up to 100 mL with distilled water, resulting in standard solutions of 0, 50, 100, 150, 250, 500 and 1000 mg/L gallic acid. These solutions were treated following the previously outlined procedure. A standard curve depicting the concentration of gallic acid versus absorbance was generated using Microsoft Excel 2019 (Microsoft Corporation, Redmond, Washington, DC, USA) yielding an R2 value of 0.9894. The computation of total polyphenols was executed by employing the equation derived from the standard curve: y = 0.0075x + 0.0728.

#### 2.2.4. Determination of Phenolic Acids by HPLC-DAD Method

The quantification and identification of phenolic acids in extracts were conducted using the method described by Martinez-Cruz and Peredes-Lopez [20] on a Shimadzu HPLC system (Shimadzu Corporation, Japan). A diode array detector from Shimadzu (Photodiode Array Detector–SPD-M30A, Shimadzu Corporation, Japan) was employed. Separation of phenolic compounds utilized a Supelco C18 column (Discovery C18, Supelco, Sigma Aldrige Poland), 5 μm, 150 mm × 4.6 mm. The separation protocol entailed a mobile phase comprising water (solvent A) and 70% aqueous acetonitrile (solvent B). Prior to analysis, all solvents underwent filtration through a 0.45 μm membrane. The gradient program for the system was as follows: 0–4 min, 0–10% B; 4–8 min, 10–15% B; 8–17 min, 15–40% B; 17–35 min, 40–100% B, and finally 3 min, 100% B. A constant flow rate of 1.0 mL/min was maintained. All reagents were sourced from the company Sigma Aldrige, Poland. Both extracts and standards were injected at a volume of 10 μL. Standard curves were constructed using the peak areas (uv. s, *y*-axis) of various concentrations (mg/mL, *x*-axis), and were expressed using linear least-squares regression equations. Each assay was performed in triplicate, and the results were presented as the mean ± standard deviation.

#### 2.2.5. Determination of Flavonoids by HPLC-DAD Method

The quantification and identification of flavonoids in extracts followed the methodology outlined by Martinez-Cruz and Peredes-Lopez [20] using a Shimadzu HPLC system (Shimadzu Corporation, Japan). A diode array detector from Shimadzu (Photodiode Array Detector–SPD-M30A, Shimadzu Corporation, Japan) was employed, and phenolic compounds were separated utilizing a Supelco C18 (Discovery C18, Supelco, Sigma Aldrige Poland), column (5 μm, 150 mm × 4.6 mm). The separation protocol involved a mobile phase consisting of 2% acetic acid in water (solvent A) and a mixture of 2% acetic acid, 30% acetonitrile, and 68% water (solvent B). Before analysis, all solvents underwent filtration through a 0.45 μm membrane. The gradient program was as follows: 0–6 min, 0–10% B; 6–10 min, 10–15% B; 10–16 min, 15–40% B; 16–30 min, 40–100% B, followed by 3 min at 100% B. The flow rate remained constant at 1.0 mL/min, and the injection volume for both extracts and standards was 10 μL. All reagents were sourced from the company Sigma Aldrige, Poland. Standard curves were generated using different concentrations (mg/mL) and their corresponding peak areas (uv. s) on the *y*-axis, with results expressed through linear least-squares regression equations. Each assay was conducted in triplicate, and the data were presented as mean ± standard deviation.

#### 2.2.6. Antioxidant Properties

##### DPPH Method

The assessment of the extracts’ antioxidant qualities was conducted via the measurement of their capacity to neutralize stable, synthetic DPPH radicals (Sigma Aldrige Poland) [21]. This procedure involves the reduction of stable DPPH radicals by the antiradical constituents within the extract, followed by the quantification of the reduction in absorbance of the radical solution subsequent to the reaction. Absorbance measurements were carried out at a wavelength of 562 nm (Shimadzu UV-1201V spectrophotometer, Shimadzu Corporation, Japan). The calculation of antiradical activity was determined using the subsequent equation:

(%) Inhibition = [(Abs control − Abs sample/Abs control)] × 100(1)

##### ABTS Method

The ability of extracts to neutralize ABTS^•+^ (Sigma Aldrige, Poland) was assessed to evaluate their antiradical properties [22]. The procedure involved the direct production of ABTS^•+^ through the oxidation of ABTS using potassium persulphate. Introduction of an antioxidant leads to the reduction of ABTS^•+^ to ABTS, resulting in a decrease in absorbance intensity (Shimadzu UV-1201V spectrophotometer, Shimadzu Corporation, Japan). Spectrophotometric measurement at 734 nm was utilized to quantify the degree of ABTS^•+^ reduction. Antiradical activity was calculated using the following equation:

(%) Inhibition = [(Abs control − Abs sample/Abs control)] × 100(2)

#### 2.2.7. Statistical Analysis

All tests were made in three repetitions. Mean values and standard deviations were calculated using the Microsoft Office Excel 2007 program. For the statistical analysis, the R Commander Program was used (Commander Program R i386 3.0.3). The analysis of the significance of the mean value differences was performed after one-way ANOVA with the post hoc Tukey’s test at α = 0.05. The analyses of the correlation between the obtained results were also calculated at α = 0.05. The antiradical properties of the extracts were also subjected to response surface analysis to determine the optimal extraction conditions. The equation used for response surface analysis is:

Y = β_0_ + β_1_X_1_ + β_2_X_2_ + β_3_X_3_ + β_11_X_1_^2^ + β_22_X_2_^2^ + β_33_X_3_^2^ + β_12_X_1_X_2_ + β_13_X_1_X_3_ + β_23_X_2_X_3_ + ϵ (3)

where: Y—optimized value, β_0_—intercept, β_1_, β_2_, β_3_—coefficients of the linear influence of variable factors, β_11_, β_22_, β_33_—coefficients of the quadratic influence of variable factors, β_12_, β_13_, β_23_—coefficients of the interaction between factors, X_1_, X_2_, X_3_—variable factors, ϵ—random error.

## 3. Results and Discussion

### 3.1. Analysis of Red clover Extracts

#### 3.1.1. Total Polyphenol Content

**Figure 1:**
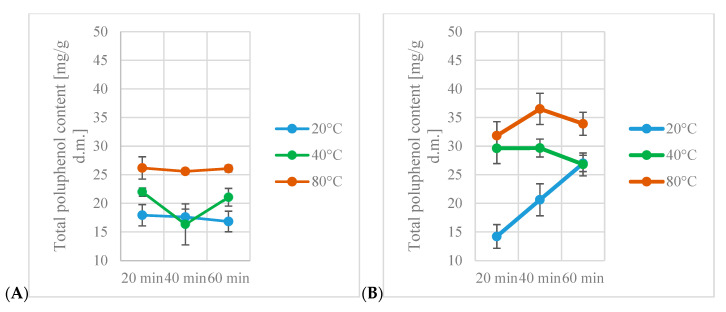

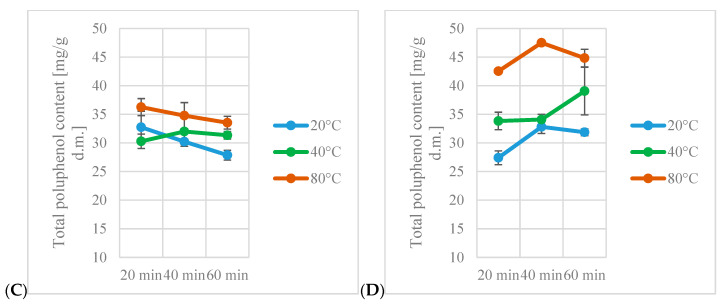
Total polyphenol content obtained in analyzed extracts: (**A**)—water, (**B**)—40% ethanol, (**C**)—60% ethanol, (**D**)—80% ethanol.

The highest amount of total polyphenols was found in the 80% ethanol extract obtained after 40 min in 80 °C (over 47 mg GAE/g d.m.), while the lowest amount was found in the 40% ethanol extract obtained after 20 min in 40 °C (around 14 mg GAE/g d.m.) (Figure 1).

In all extracts, the influence of individual factors on the total polyphenol content was found. Increasing the temperature, time or ethanol content in the extracts resulted in an increase in the total polyphenol content in the obtained extracts. Increasing the values of all factors resulted in an increase in the total polyphenol content. This effect was greater than the effect of increasing the value of just one factor. The only exception was the interaction between ethanol content, extraction time and extraction temperature in the 40% and 60% ethanol extracts. It was found that increasing the extraction temperature resulted in an increase in the total polyphenol content, while extending the extraction time resulted in an increase in the total polyphenol content only in the cases of the 40% and 80% ethanol extracts. Of all the ethanol extracts tested, it was noticed that the smallest differences (regardless of the temperature and extraction time used) in the amount of polyphenols obtained were in the case of the 40% ethanol extract. It should be noted, however, that when using a 60% ethanol solution, the difference between the extreme values was at a similar level, unlike most other ethanol solutions.

Akbarizbam et al. [23] obtained a higher amount of total polyphenols using 99.6% ethanol extracts. Total polyphenol content in their extracts reached over 58 mg GAE/g d.m. Esmaeili et al. [24] obtained extracts by using organic solvents, such as methanol, n-hexane, ethyl acetate and chloroform. The highest total polyphenol content was found in the 95% methanol extract. Total polyphenol content reached 46 mg GAE/g d.m. in this extract. This value is close to the value of the 80% ethanol extract obtained after 40 min at 80 °C (around 47.5 mg GAE/g d.m.). Tava et al. [25] investigated the influence of the place of harvest and type of cultivation on total polyphenol content in different variates of clovers, including red clover. They used 80% methanol extracts obtained at 40 °C. Depending on the type of cultivation, total polyphenol content ranged from 46.9 to 72.9 mg GAE/g d.m. Similar values were found in *Trifolium pratense* subsp. *nivale* (47 and 49 mg GAE/g d.m.). The other two red clover extracts had total polyphenol content of 69 and 73 mg GAE/g d.m. The higher amounts of total polyphenols obtained by Tava et al. [25] might be a result of the use of high pressure in extraction and different red clover variates.

Kazlauskaite et al. [26] extracted red clover using water and 50% ethanol. For both types of extract, total polyphenol content was higher than the amounts in the current research. The total polyphenol content of the 50% ethanol extracts reached over 40 mg GAE/g d.m. Only some of the 80% ethanol extracts in the current research contained over 40 mg GAE/g d.m. and the water extracts contained below 26 mg GAE/g d.m. Meanwhile, the water extracts obtained by Kazlauskaite et al. [26] contained over 30 mg GAE/g d.m. Chiriac et al. [27] analyzed total polyphenol content in water extracts of red clover. Their total polyphenol contents ranged from 15 to 20 mg GAE/g d.m. and are similar to those obtained in the current research (14–26 mg GAE/g d.m.).

Statistical analysis of all extracts confirmed the effect of all factors and their interactions on total polyphenol content. Increases in temperature, time or ethanol content in extracts resulted in an increase in total polyphenol content in the obtained extracts. Interactions between all factors also had a significant impact on total polyphenol content. Increasing values of all factors caused an increase in total polyphenol content. This effect was higher than the effect of increasing the value of only one factor. The only exception was the interaction between ethanol content, extraction time and extraction temperature in 40% and 60% ethanol. Statistical analysis of extract groups showed that increasing extraction temperature resulted in an increase in total polyphenol content, while an increase in extraction time resulted in an increase of total polyphenol content only for the 40% and 80% ethanol extracts.

For water extracts, total polyphenol content decreased after 40 min of extraction and increased after 60 min, reaching a lower value than after 20 min of extraction. Total polyphenol content of 60% ethanol extracts decreased with an increase in extraction time. Interactions between extraction time and temperature also had an impact on total polyphenol content. An increase in the values of these parameters resulted in an increase of total polyphenol content.

**Figure 2:**
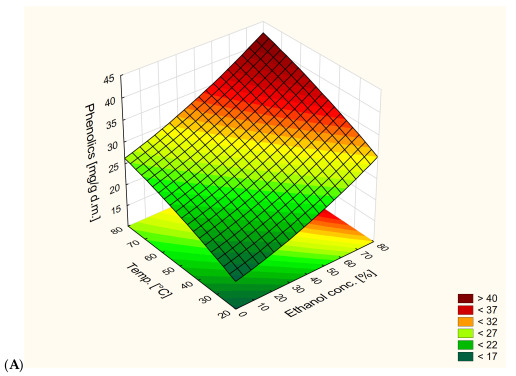

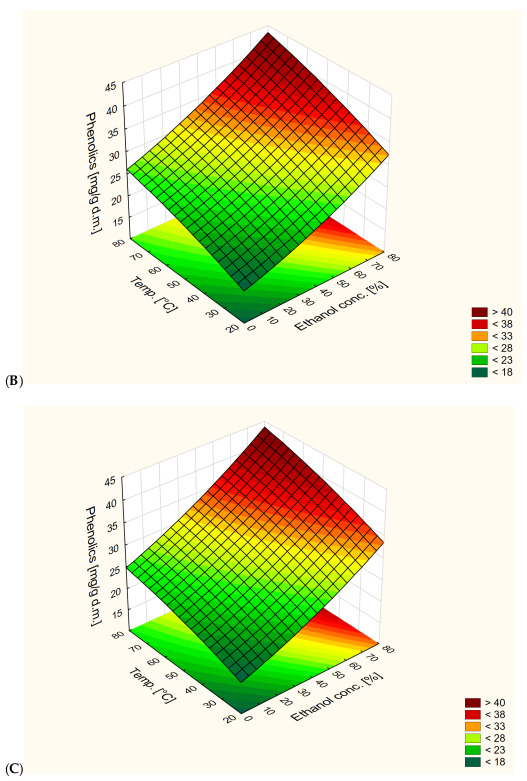
Response surface plot of the dependence of total polyphenol content on extraction temperature and ethanol content in extraction solution at different extraction times ((**A**)—20 min, (**B**)—40 min, (**C**)—60 min).

Figure 2 shows the effect of extraction temperature and ethanol content on total polyphenol content for different extraction times. The highest values were obtained for 60 min of extraction, while the lowest values were obtained for 20 min of extraction. Both extraction temperature and ethanol content had an impact on total polyphenol content. Increases in temperature and ethanol content resulted in an increase in polyphenol content. Based on the obtained graphs, the best conditions for the extraction of polyphenols are 80% ethanol solvent, 80 °C and 60 min of extraction.

The results depicted in Figure 2 shed light on the intricate relationship between extraction parameters and total polyphenol content. It is evident that both temperature and ethanol concentration play pivotal roles in determining the efficiency of polyphenol extraction. The observed trend, wherein higher temperatures and ethanol concentrations correlate with elevated polyphenol content, underscores the importance of these factors in optimizing the extraction process.

Similarly, as in the case of total polyphenols, the highest content of flavonoids, especially daidzein (5.13 mg/g d.m.) and genistein (7.24 mg/g d.m.), was observed when extracted with 80% ethanol at 80 °C for 40 min (Table 1). In general, increasing ethanol, temperature and extraction time increased the content of individual flavonoids in the extracts. However, not all differences were statistically significant. Only a slight and not statistically significant decrease was observed in the case of extraction with 80% ethanol at 80 °C for 60 min compared to extraction for 40 min under the same conditions. In the case of aqueous extracts, the content of both daidzein and genistein decreased after 40 min and increased after 60 min, reaching a value lower than after 20 min. The labeled phenolic acids in some extracts were not detected (Table 2). In general, their content was at a low level. The highest amount of chlorogenic acid was found after a 40 min extraction with 60% ethanol at 40 °C (0.25 mg/g d.m.). There was no effect of the type of solvent, extraction time or temperature used on the content of phenolic acids in the extracts (the differences were not statistically significant).

#### 3.1.2. DPPH Free Radical Assay

**Figure 3:**
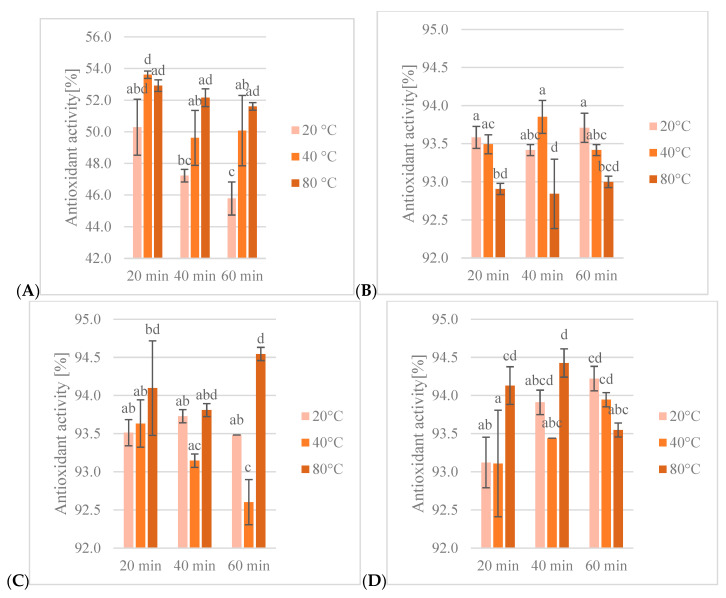
Antioxidant activity in DPPH free radical assay [%] of obtained extracts: (**A**)—water, (**B**)—40% ethanol, (**C**)—60% ethanol, (**D**)—80% ethanol. Lowercase letters (a–d) indicate statistically significant differences in individual groups (*p* < 0.05).

DPPH^•^ reduction assay showed a 92.6–94.5% reduction in free radicals for ethanol extracts and 45.8–53.6% reduction in free radicals for water extracts (Figure 3). The highest antioxidant activity of ethanol extracts was observed in the 60% ethanol extract, obtained after 60 min of extraction at 80 °C (94.5%), and the lowest activity was observed in the 60% ethanol extract obtained after 60 min of extraction at 20 °C (92.6%). In water extracts, the highest activity was observed in the extract obtained after 20 min of extraction at 40 °C (53.6%) and the lowest was observed in the extract obtained after 60 min at 20 °C.

The obtained results of antioxidant activity in DPPH^•^ assay (46–54%) are higher than the results obtained by Chiriac et al. [27], whose results ranged between 15% and 45%. Esmaeili et al. [24] obtained the best results in ethyl acetate extracts. Similar results were obtained for methanol extracts, in which antioxidant activity reached around 70%. Kaurinovic et al. [28] studied antioxidant activity by determining half-maximal inhibitory concentration (IC_50_). The best results were obtained for water extracts, in which IC_50_ values were similar to synthetic antioxidant compounds. Kazlauskaite et al. [26] compared water and 50% ethanol extracts. The highest antioxidant activities were obtained for ethanol extract, but some water extracts showed similar activity to ethanol extracts. The results obtained in this research also showed the higher antioxidant activity of ethanol extracts. Similar results were obtained in the study of Çölgeçen et al. [29].

Statistical analysis of extracts showed a significant impact of ethanol content on antioxidant activity in DPPH^•^ reduction assay but showed no differences between ethanol extracts. Also, both extraction time and temperature had a significant impact on antioxidant activity and interactions between factors, except the interaction between the parameters of extraction time and temperature. An increase in extraction temperature resulted in higher antioxidant activity, while an increase in extraction time had the opposite effect. In the analysis of groups of extracts, the effects of different extraction conditions led to different outcomes. For water extracts, increasing temperature resulted in lower activity, but for 40% ethanol extracts it was higher. For 60% and 80% ethanol extracts, antioxidant activity decreased after heating to 40 °C and increased after heating to 80 °C to a higher value than the extracts obtained at 20 °C. An interaction between extraction time and temperature was observed in ethanol extracts. Extraction time only had a significant effect for water extracts and 80% ethanol extracts. The antioxidant activity of water extracts decreased with an increase in extraction temperature. For 80% ethanol extracts, after 40 min, antioxidant activity increased and then decreased after 60 min.

**Figure 4:**
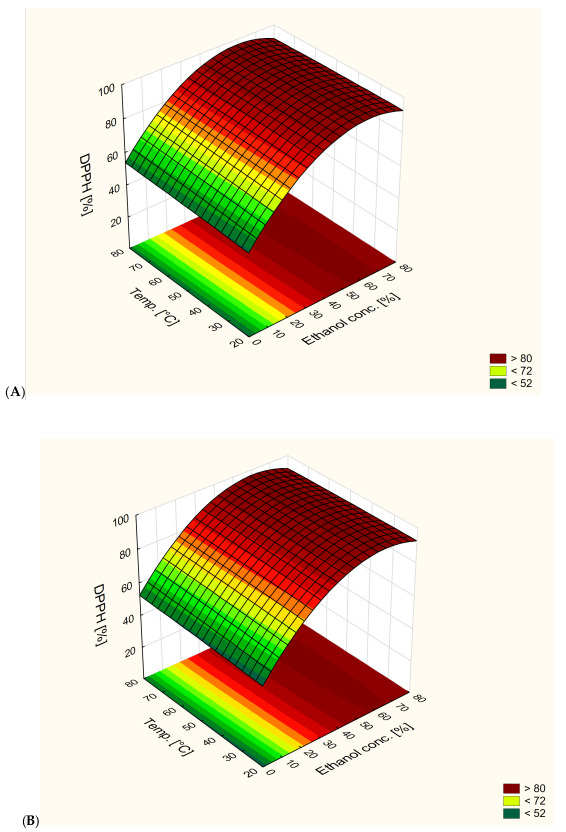

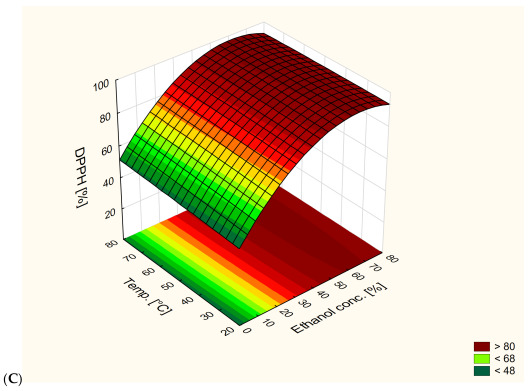
Response surface plot of the dependence of antioxidant activity against DPPH radicals on extraction temperature and ethanol content in extraction solutions at different extraction times ((**A**)—20 min, (**B**)—40 min, (**C**)—60 min).

Figure 4 shows the effect of extraction temperature and ethanol content on antioxidant activity at different extraction times. Ethanol concentration had a significant effect on antioxidant activity. The highest values were obtained for solvents containing around 65% of ethanol at all extraction times. For all extraction times, the optimal ethanol content was around 60–70%; however, a decrease in the optimal range of ethanol concentration was observed in extracts obtained after 40 min of extraction. For those extracts, the optimal range was 60–65%. A decrease in antioxidant activity was observed with an increase in ethanol concentration in solvents above 70%. Time also had a significant effect on antioxidant activity. With an increase in extraction time, the ethanol concentration required to obtain extracts with similar activity also increased. Lower extraction times allowed extracts with similar antioxidant activities and lower ethanol content to be obtained.

The findings presented in Figure 4 highlight the significant influence of extraction temperature and ethanol concentration on the antioxidant activity of the extracts. Specifically, it is evident that ethanol concentration plays a pivotal role in modulating antioxidant activity, with the highest values observed at approximately 65% ethanol content across all extraction times. This suggests that the presence of ethanol within this optimal range facilitates the extraction of antioxidant compounds from the sample matrix. Moreover, the results indicate a nuanced relationship between ethanol concentration and extraction time, wherein the optimal ethanol content for maintaining antioxidant activity shifts with varying extraction durations. Notably, while the optimal range generally falls within 60–70% ethanol concentration, a reduction in this range is observed for extracts obtained after 40 min of extraction, where the optimal ethanol content ranges from 60% to 65%. This suggests a temporal dependency on the ethanol concentration required to achieve maximum antioxidant activity, potentially reflecting changes in the solubility and diffusion kinetics of antioxidant compounds over the course of extraction.

Furthermore, the impact of extraction time on antioxidant activity is underscored by the observed trend wherein longer extraction durations necessitate higher ethanol concentrations to achieve comparable antioxidant activity. Prolonged extraction times may lead to a diminished yield of antioxidant compounds per unit volume of solvent, necessitating higher ethanol concentrations to maintain consistent activity levels.

#### 3.1.3. ABTS Radical Assay

**Figure 5:**
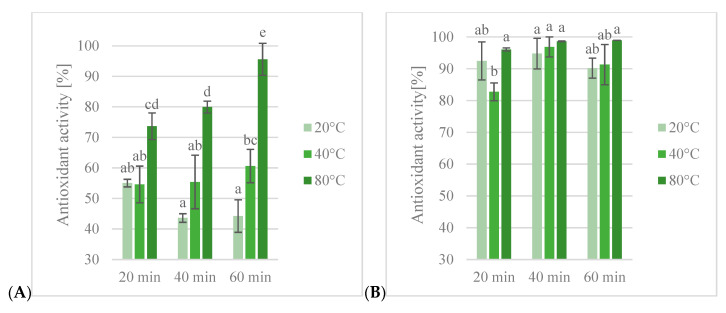

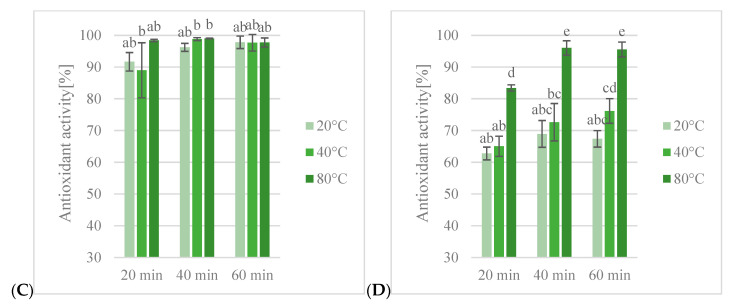
Antioxidant activity in ABTS radical assay [%] of obtained extracts: (**A**)—water, (**B**)—40% ethanol, (**C**)—60% ethanol, (**D**)—80% ethanol. Lowercase letters (a–e) indicate statistically significant differences in individual groups (*p* < 0.05).

The highest antioxidant activity was observed in the 60% ethanol extract obtained at 80 °C after 40 min (99.0%), while the lowest was observed in the water extract obtained at 20 °C after 40 min (43.6%) (Figure 5). Water extracts obtained at 80 °C after 40 min and in 40 °C after 60 min showed similar activity as 80% ethanol extracts. No significant difference was observed between the water extract obtained at 80 °C after 60 min and 40% and 60% ethanol extracts. There was no significant difference between the 40% and 60% ethanol extracts, except for the 40% ethanol extract obtained at 40 °C after 20 min.

Results obtained by Kazlauskaite et al. [26] showed no significant difference in antioxidant activity between water extracts and 50% ethanol extracts. In the current research, some water extracts showed activity similar to ethanol extracts. Mikulic et al. [30] reached similar conclusions.

All extraction parameters and their interactions had significant effect on antioxidant activity. The effect of ethanol content and its interactions with other parameters caused an increase in activity, and then a decrease after reaching 60% ethanol content in solvent.

The exceptions to this were extracts obtained at 20 °C after 20 min and at 80 °C after 40 and 60 min, where the maximum activity was obtained in 40% ethanol extracts. An increase in antioxidant activity was also observed with an increase in extraction time and temperature. Statistical analysis of groups of extracts showed the effect of extraction time on antioxidant activity. The antioxidant activity of water extracts decreased after 40 min of extraction and increased after 60 min. In the case of ethanol extracts, antioxidant activity increased after 40 min, and for 80% ethanol extracts, it also increased after 60 min. For 40% and 60% ethanol extracts, it decreased. The 60% ethanol extracts were the only group that did not show a significant effect of temperature on antioxidant activity. The antioxidant activity of 80% ethanol extracts increased with an increase in extraction time, but for 40% ethanol extracts it decreased after 40 min and increased after 60 min. The interaction between extraction time and temperature had a significant effect on the antioxidant activity of water and 40% ethanol extracts. For water extracts, both an increase in temperature and time led to an increase in antioxidant activity. In 40% ethanol extracts obtained at 40 °C, after 20 min antioxidant activity dropped, but after 40 and 60 min of extraction it increased.

**Figure 6:**
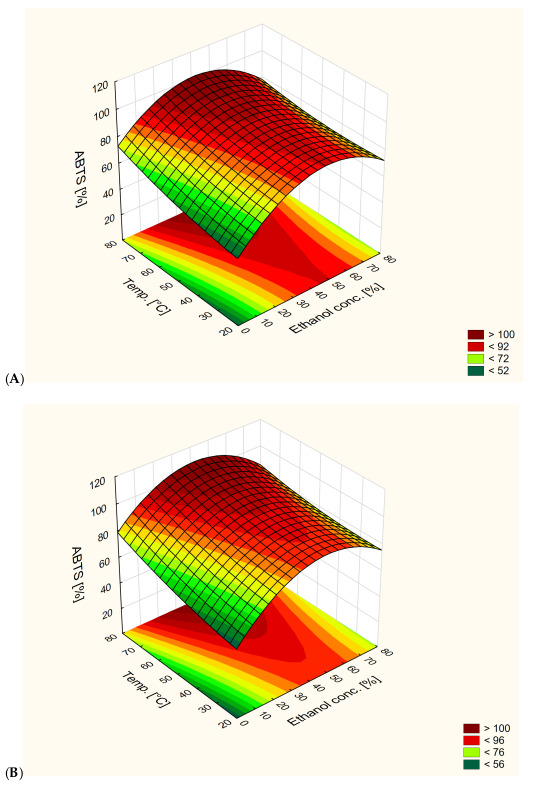

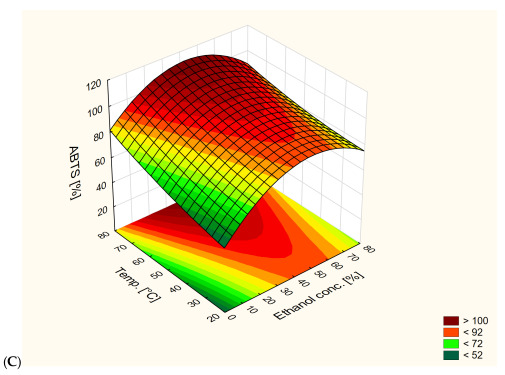
Response surface plot of the dependence of antioxidant activity against ABTS radicals on extraction temperature and ethanol content in extraction solutions at different extraction times ((**A**)—20 min, (**B**)—40 min, (**C**)—60 min).

Figure 6 shows the effect of extraction temperature and ethanol content on antioxidant activity at different extraction times. All parameters had a significant effect on antioxidant activity. The highest values of antioxidant activity were obtained for 50% ethanol extracts. An increase in ethanol concentration in solvents above 50% led to a decrease in antioxidant activity. The ability to obtain extracts able to reach 100% ABTS^•+^ reduction was observed for all extraction times.

The results presented in Figure 6 show a clear effect of extraction temperature and ethanol content on the antioxidant activity of extracts at different extraction times. According to other studies, both parameters significantly affect the antioxidant potential of the extracts. Notably, the highest antiradical activity was consistently observed in extracts obtained using 50% ethanol as the solvent. This statement is consistent with research by other scientists emphasizing the importance of ethanol concentration in optimizing antioxidant extraction [31,32].

The highest antioxidant activities were observed In extracts obtained after 60 min of extraction. After 20 min of extraction, extracts could possess 100% ABTS radical reduction at 80 °C and an ethanol concentration of around 50%. After 40 min of extraction, the same value could be obtained at 70 °C, and after 60 min at 60 °C, with the same ethanol concentrations. With an increase in extraction time, the extraction time required to obtain extracts with similar activities decreased. In addition, 20% ethanol extracts showed similar activities to 80% ethanol extracts obtained in the same conditions. Increasing ethanol content from 20% or decreasing from 80% in order to obtain 50% ethanol content led to obtaining extracts with the same antioxidant activities. It is possible to obtain extracts with the same antioxidant activity through changes in extraction conditions.

The possibility of obtaining similar antiradical activities using extracts with different ethanol concentrations highlights the flexibility of the extraction process. For example, extracts containing 20% ethanol showed antiradical activity comparable to extracts containing 80% ethanol when extracted under similar conditions. This suggests that regulation of ethanol content can be used to modulate antioxidant activity without compromising effectiveness, which is also confirmed by the work of other researchers [31].

### 3.2. Enriched Drinks Analysis

Enriched blackcurrant beverages had higher total polyphenol content than the control beverage (Table 3). The values depended on the size of the additive, and the effect of the type of extract was not significant. Statistical analysis showed significant differences in total polyphenol content between the enriched beverages and the control beverage, but only for an additive of 5%.

Enriched blackcurrant beverages had higher total polyphenol content than the control beverage (Table 4). The values depended on the size of the additive, and the effect of the type of extract was not significant. Statistical analysis showed significant differences in total polyphenol content between the enriched beverages and the control beverage, but only for an additive of 5%.

**Figure 7:**
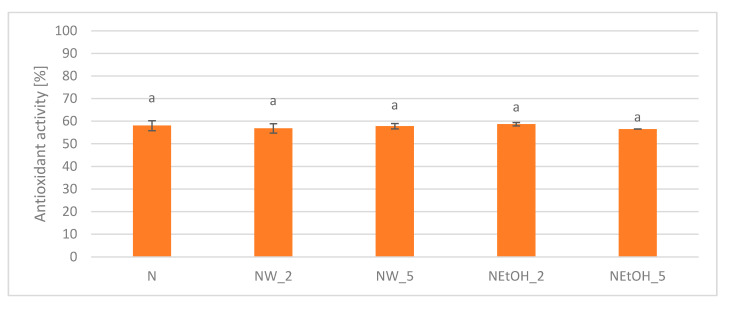
Antioxidant activity of enriched drinks compared to control drink in DPPH free radical assay. Lowercase letters indicate statistically significant differences in individual groups (*p* < 0.05).

Antioxidant activity in the DPPH free radical assay did not show significant differences between enriched drinks and the control drink. For all samples, its value ranged from 56.5% to 58.7% (Figure 7).

**Figure 8:**
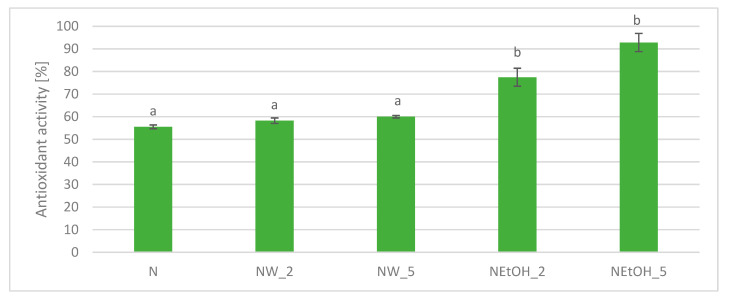
Antioxidant of enriched drinks compared to control drink in ABTS radical assay. Lowercase letters indicate statistically significant differences in individual groups (*p* < 0.05).

Antioxidant activity against ABTS radicals (Figure 8) was different for drinks enriched with ethanol extracts. Those drinks showed higher activity that increased with addition level. Although the 5% addition level showed higher antioxidant activity, statistical analysis did not show any significant differences between 2% and 5% addition levels. Antioxidant activity of control drinks was above 55%, but enriched drinks reached 77.5% and 92.8% antioxidant activity for the 2% enriched drink and 5% enriched drink, respectively. Antioxidant activity of water extract-enriched drinks reached around 60%. Statistical analysis showed that the addition of extracts had an impact on the antioxidant activity in the presence of ABTS cation radicals in the case of ethanol extracts.

## 4. Conclusions

This study showed a significant effect of temperature and extraction time, as well as the ethanol content of the extraction solution, on the antiradical properties of the extracts obtained. For all factors, an increase in polyphenol content was observed with an increase in the values of these factors. The highest polyphenol contents were characterized by 80% ethanol extracts, and the lowest by aqueous extracts. It was observed that conducting extraction for 20 min allowed extracts with stronger antioxidant properties in the presence of DPPH radicals to be obtained, while increasing the duration of the extraction caused a decrease in favor of an increase in activity in the presence of ABTS radicals. It was also observed that there was no effect of increasing the total polyphenol content on antioxidant activity. The 80% ethanol extracts, despite their higher polyphenol content, had similar antioxidant activity, and even lower activity in the presence of ABTS radicals, as the 40% and 60% ethanol extracts.

Analysis of the response surface made it possible to determine the optimal extraction conditions depending on the selected factor. In order to obtain extracts with the highest content of total polyphenols, the value of all factors had to be increased. However, this led to a decrease in antioxidant activity against both analyzed radicals. For the DPPH radical, the best activity could be obtained when extracted for 20 min in a solution of about 65% at low temperatures. For the ABTS radical, the best antioxidant activity could be obtained when extracted for 60 min at 80 °C using a solution of about 50% ethanol. Using a solution of about 60% ethanol when extracted for 60 min at 80 °C, on the other hand, produced an extract with simultaneous high antioxidant activity against both radicals.

It was found that enriched blackcurrant beverages had a higher content of total polyphenols than the control beverage. The values depended on the size of the additive, and the effect of the type of extract was insignificant. The clover-specific polyphenols daidzein and genistein were also found. The content of these compounds was dependent on the amount of extract added, as well as the type of solvent. Attempts to enrich beverages and other foods with ethanol extracts from red clover require further research.

## Figures and Tables

**Table 1:** Content of selected flavonoids in the tested extracts.

Extract	Daidzein	Genistein	Quercetin	Rutin	Kaempferol
	mg/g d.m.				
Water					
20 min					
20 °C	2.45 (±0.05) a	2.92 (±0.23) a	0.73 (±0.01) a	0.24 (±0.01) a	* nd
40 °C	2.54 (±0.04) a	3.13 (±0.01) ab	0.67 (±0.03) a	0.23 (±0.01) a	nd
80 °C	2.75 (±0.03) a	3.24 (±0.03) b	0.78 (±0.03) a	0.25 (±0.02) a	0.09 (±0.01) a
40 min					
20 °C	1.78 (±0.03) b	2.13 (±0.06) a	0.79 (±0.00) a	0.25 (±0.00) a	nd
40 °C	1.89 (±0.07) b	2.23 (±0.04) a	0.87 (±0.00) a	0.27 (±0.00) a	nd
80 °C	1.90 (±0.02) b	2.43 (±0.01) a	0.84 (±0.01) a	0.24 (±0.01) a	0.09 (±0.03) a
60 min					
20 °C	2.12 (±0.09) a	2.71 (±0.02) a	0.87 (±0.03) a	0.23 (±0.01) a	nd
40 °C	2.16 (±0.06) a	2.98 (±0.01) ab	0.88 (±0.01) a	0.26 (±0.00) a	nd
80 °C	2.45 (±0.02) a	3.12 (±0.01) ab	0.85 (±0.01) a	0.24 (±0.00) a	0.09 (±0.00) a
Ethanol 40%					
20 min					
20 °C	2.12 (±0.03) a	2.87 (±0.00) a	1.12 (±0.04) a	0.27 (±0.01) a	0.10 (±0.00) a
40 °C	2.42 (±0.02) a	3.01 (±0.03) ab	1.15 (±0.03) a	0.26 (±0.01) a	0.11 (±0.00) a
80 °C	2.67 (±0.01) a	3.15 (±0.02) ab	1.17 (±0.01) a	0.28 (±0.03) a	0.11 (±0.01) a
40 min					
20 °C	2.69 (±0.04) a	3.15 (±0.07) ab	1.18 (±0.02) a	0.25 (±0.01) a	0.10 (±0.00) a
40 °C	2.70 (±0.06) a	3.24 (±0.05) b	1.16 (±0.01) a	0.25 (±0.01) a	0.11 (±0.00) a
80 °C	2.98 (±0.05) a	3.26 (±0.03) b	1.19 (±0.03) a	0.26 (±0.02) a	0.11 (±0.01) a
60 min					
20 °C	2.72 (±0.05) a	3.19 (±0.02) ab	1.16 (±0.01) a	0.26 (±0.01) a	0.11 (±0.00) a
40 °C	2.87 (±0.03) a	3.30 (±0.01) b	1.25 (±0.00) a	0.25 (±0.01) a	0.12 (±0.00) a
80 °C	3.10 (±0.03) ac	3.46 (±0.08) b	1.27 (±0.03) a	0.27 (±0.01) a	0.11 (±0.01) a
Ethanol 60%					
20 min					
20 °C	2.89 (±0.09) a	3.25 (±0.01) b	1.18 (±0.06) a	0.27 (±0.02) a	0.10 (±0.01) a
40 °C	2.97 (±0.02) a	3.37 (±0.01) b	1.23 (±0.03) a	0.27 (±0.02) a	0.11 (±0.01) a
80 °C	3.17 (±0.01) ac	3.65 (±0.04) b	1.25 (±0.03) a	0.28 (±0.03) a	0.11 (±0.02) a
40 min					
20 °C	2.90 (±0.04) a	3.45 (±0.02) b	1.18 (±0.01) a	0.27 (±0.00) a	0.11 (±0.01) a
40 °C	3.12 (±0.03) ac	3.49 (±0.03) b	1.24 (±0.01) a	0.28 (±0.02) a	0.12 (±0.01) a
80 °C	3.34 (±0.03) c	3.87 (±0.00) bc	1.25 (±0.01) a	0.30 (±0.01) a	0.12 (±0.02) a
60 min					
20 °C	3.98 (±0.04) d	3.76 (±0.02) bc	1.21 (±0.02) a	0.22 (±0.01) a	0.09 (±0.03) a
40 °C	4.34 (±0.03) d	3.54 (±0.01) b	1.23 (±0.02) a	0.26 (±0.01) a	0.10 (±0.02) a
80 °C	4.48 (±0.01) d	4.12 (±0.06)c	1.23 (±0.03) a	0.25 (±0.03) a	0.09 (±0.02) a
Ethanol 80%					
20 min					
20 °C	4,35 (±0.01) d	5.23 (±0.05) d	1.19 (±0.03)	0.22 (±0.02) a	0.10 (±0.00) a
40 °C	4.64 (±0.04) d	5.78 (±0.01) d	1.24 (±0.03) a	0.23 (±0.01) a	0.10 (±0.01) a
80 °C	4.73 (±0.04) d	5.92 (±0.02) d	1.23 (±0.03) a	0.24 (±0.00) a	0.11 (±0.01) a
40 min					
20 °C	4.60 (±0.06) d	6.15 (±0.01) d	1.23 (±0.01) a	0.23 (±0.00) a	0.11 (±0.01) a
40 °C	4.89 (±0.03) d	6.65 (±0.03) d	1.23 (±0.02) a	0.25 (±0.00) a	0.10 (±0.01) a
80 °C	5.11 (±0.02) e	7.24 (±0.03) e	1.25 (±0.02) a	0.26 (±0.01) a	0.10 (±0.01) a
60 min					
20 °C	4.68 (±0.02) d	6.24 (±0.04) d	1.20 (±0.05) a	0.24 (±0.02) a	0.11 (±0.01) a
40 °C	4.92 (±0.03) d	6.76 (±0.02) d	1.20 (±0.05) a	0.26 (±0.02) a	0.12 (±0.01) a
80 °C	5.00 (±0.03) de	7.14 (±0.01) e	1.20 (±0.03) a	0.27 (±0.03) a	0.12 (±0.01) a

* nd: not detected. Lowercase letters in columns indicate statistically significant differences in individual groups. Differences between mean values marked with different letters in the line are statistically significant (*p* < 0.05).

**Table 2:** Content of selected phenolic acids in the tested extracts.

Extract	Caffeic Acid	Chlorogenic Acid	Gallic Acid
	mg/g d. m.		
Water			
20 min			
20 °C	nd	nd	nd
40 °C	nd	nd	nd
80 °C	0.09 (±0.01)	0.11 (±0.01) a	0.09 (±0.00) a
40 min			
20 °C	nd	0.09 (±0.00) a	nd
40 °C	nd	0.10 (±0.01) a	nd
80 °C	0.09 (±0.02) a	0.10 (±0.01) a	0.09 (±0.00) a
60 min			
20 °C	0.08 (±0.01) a	0.11 (±0.01) a	nd
40 °C	0.09 (±0.00) a	0.10 (±0.02) a	nd
80 °C	0.10 (±0.01) a	0.10 (±0.00) a	0.11 (±0.00) a
Ethanol 40%			
20 min			
20 °C	nd	nd	0.10 (±0.02) a
40 °C	nd	0.21 (±0.00) a	0.10 (±0.01) a
80 °C	0.09 (±0.03) a	0.22 (±0.01) a	0.12 (±0.01) a
40 min			
20 °C	0.09 (±0.00) a	0.10 (±0.01) a	0.09 (±0.01) a
40 °C	0.10 (±0.00) a	0.19 (±0.01) a	0.09 (±0.01) a
80 °C	0.10 (±0.00) a	0.22 (±0.00) a	0.12 (±0.01) a
60 min			
20 °C	0.10 (±0.00) a	0.17 (±0.01) a	0.10 (±0.00) a
40 °C	0.10 (±0.00) a	0.20 (±0.01) a	0.11 (±0.00) a
80 °C	0.11 (±0.01) a	0.22 (±0.02) a	0.11 (±0.01) a
Ethanol 60%			
20 min			
20 °C	0.10 (±0.00) a	0.19 (±0.01) a	0.09 (±0.00) a
40 °C	0.10 (±0.00) a	0.22 (±0.00) a	0.10 (±0.01) a
80 °C	0.11 (±0.01) a	0.22 (±0.00) a	0.11 (±0.00) a
40 min			
20 °C	0.10 (±0.02) a	0.20 (±0.00) a	0.11 (±0.01) a
40 °C	0.11 (±0.03) a	0.25 (±0.00) a	0.11 (±0.01) a
80 °C	0.12 (±0.01) a	0.23 (±0.01) a	0.12 (±0.01) a
60 min			
20 °C	0.10 (±0.00) a	0.19 (±0.00) a	0.10 (±0.01) a
40 °C	0.10 (±0.00) a	0.21 (±0.03) a	0.10 (±0.01) a
80 °C	0.10 (±0.02) a	0.22 (±0.01) a	0.11 (±0.01) a
Ethanol 80%			
20 min			
20 °C	0.11 (±0.01) a	0.20 (±0.01) a	0.12 (±0.00) a
40 °C	0.10 (±0.01) a	0.23 (±0.01) a	0.12 (±0.00) a
80 °C	0.11 (±0.01) a	0.22 (±0.01) a	0.11 (±0.01) a
40 min			
20 °C	0.12 (±0.00) a	0.23 (±0.00) a	0.10 (±0.01) a
40 °C	0.11 (±0.01) a	0.23 (±0.00) a	0.11 (±0.01) a
80 °C	0.11 (±0.01) a	0.23 (±0.01) a	0.11 (±0.02) a
60 min			
20 °C	0.12 (±0.00) a	0.21 (±0.00) a	0.12 (±0.01) a
40 °C	0.13 (±0.02) a	0.22 (±0.00) a	0.12 (±0.01) a
80 °C	0.15 (±0.01) a	0.23 (±0.01) a	0.13 (±0.01) a

nd: not detected. Lowercase letters in columns indicate statistically significant differences in individual groups (*p* < 0.05).

**Table 3:** Total polyphenol content of enriched blackcurrant drinks compared to control sample.

Sample	Addition Level [%]	Total Polyphenol Content [mg GAE/100 cm^3^ Drink]	SD
C	-	110.35 ^a^	6.60
EW	2	95.61 ^a^	3.05
EW	5	172.49 ^b^	5.46
EEtOH	2	96.62 ^a^	1.37
EEtOH	5	183.10 ^b^	1.95

Lowercase letters in the column indicate statistically significant differences within one group. The differences between the mean values marked with different letters in the column are statistically significant (*p* < 0.05).

**Table 4:** Content of flavonoids and phenolic acids in blackcurrant drinks.

Sample	Daidzein	Genistein	Quercetin	Rutin	Kaempferol	Caffeic Acid	Chlorogenic Acid	Gallic Acid
	[mg/100 cm^3^]							
C	nd *	nd	1.07 (±0.23) ^a^	1.02 (±0.06) ^a^	0.92 (±0.05) ^a^	0.54 (±0.04) ^a^	0.92 (±0.03) ^a^	0.32 (±0.02) ^a^
EW 2%	5.32 (±0.92) ^A^	7.45 (±0.95) ^A^	2.70 (±0.72) ^bA^	2.51 (±0.09) ^bA^	0.98 (±0.02) ^aA^	0.57 (±0.93) ^aA^	0.92 (±0.02) ^aA^	0.32 (±0.02) ^aA^
EW 5%	6.67 (±1.02) ^A^	8.67 (±0.97) ^A^	0.89 (±0.62) ^bA^	2.67 (±0.68) ^bA^	0.95 (±0.02) ^aA^	0.54 (±0.2) ^aA^	0.96 (±0.10) ^aA^	0.32 (±0.01) ^aA^
EEtOH 2%	12.02 (±1.12) ^B^	14.98 (±0.12) ^B^	3.08 (±0.73) ^bA^	2.89 (±0.57) ^bA^	0.99 (±0.03) ^aA^	0.59 (±0.04) ^aA^	0.95 (±0.06) ^aA^	0.32 (±0.03) ^aA^
EEtOH 5%	21.76 (±1.23) ^C^	27.78 (±1.15) ^C^	3.10 (±0.23) ^bA^	2.79 (±0.16) ^bA^	1.07 (±0.204) ^aA^	0.61 (±0.03) ^aA^	0.97 (±0.01) ^aA^	0.32 (±0.201) ^aA^

* nd: not detected. Lowercase letters in columns indicate statistically significant differences from the control sample. Differences between mean values marked with different letters in the line are statistically significant (*p* < 0.05). Capital letters in the columns indicate statistical differences between the types of added extracts. Differences between mean values marked with different letters in the line are statistically significant (*p* < 0.05).

## Data Availability

The data presented in this study are publicly available.
